# A conceptual disease model for quality of life in mitochondrial disease

**DOI:** 10.1186/s13023-022-02411-9

**Published:** 2022-07-15

**Authors:** Kim F. E. van de Loo, Nander T. van Zeijl, José A. E. Custers, Mirian C. H. Janssen, Christianne M. Verhaak

**Affiliations:** 1 Department of Medical Psychology, Radboud Center for Mitochondrial Medicine, Amalia Children’s Hospital, Radboud Institute for Health Sciences, Radboud University Medical Center, Geert Grooteplein Zuid 10, PO Box 9101, 6500 HB Nijmegen, The Netherlands; 2 Department of Internal Medicine, Radboud Center for Mitochondrial Medicine, Radboud Institute for Molecular Life Sciences, Radboud University Medical Center, Geert Grooteplein Zuid 10, PO Box 9101, 6500 HB Nijmegen, The Netherlands

**Keywords:** Mitochondrial disease, Quality of life, Mental health, Cognitive functioning, Depressive symptoms, Fatigue, Societal participation, Disease manifestation, Wilson and Cleary model

## Abstract

**Background:**

Previous studies in patients with a mitochondrial disease (MD) highlight the high prevalence of cognitive impairments, fatigue, depression, and a lower quality of life (QoL). The relationship with biological and physiological factors remains complex. The aim of this study is to investigate the status of and interrelationships between biological and physiological functioning, cognitive functioning as well as fatigue, depression, societal participation, health perceptions, and QoL, by using the Wilson and Cleary conceptual disease model, adapted to MD.

**Methods:**

Patients with a genetically confirmed MD were included. The following health concepts in MD were investigated according to the conceptual model: (1) Biological and physiological: disease manifestation (Newcastle Mitochondrial Disease Adult Scale), (2) Symptom status: cognitive functioning, patient reported fatigue and depressive symptoms, (3) Functional health: societal participation, (4) Patient reported health perceptions, and (5) Overall QoL. Data were compared to healthy normative data and/or data from other patient groups. Correlations as well as a hierarchical regression analysis were performed to assess the relations between the different levels of health concepts in the conceptual model.

**Results:**

Of the 95 included patients, 42% had a severe disease manifestation. Comparable or worse than normative data and other patient groups, 35% reported cognitive impairments, 80% severe fatigue, and 27% depressive symptoms. Patients experienced impairments in societal participation and QoL. Disease manifestation was significantly correlated with cognitive functioning, societal participation, physical functioning and overall QoL, but not with fatigue or depressive symptoms. Almost all outcome measures regarding functional health, health perceptions and QoL were correlated with symptom status variables. Overall QoL was significantly predicted by fatigue and physical functioning.

**Conclusions:**

Symptom status is related to the functional health, health perceptions and QoL in patients with MD. Moreover, fatigue and physical functioning are important contributors to the overall QoL of MD patients. In order to provide adequate patient care it is important to have a broad view on patients’ functioning, not only by providing a proper clinical assessment, but also to screen for symptom status; cognitive functioning, fatigue and depression.

**Supplementary Information:**

The online version contains supplementary material available at 10.1186/s13023-022-02411-9.

## Introduction

The term mitochondrial disease (MD) encompasses a large and heterogeneous group of disorders and syndromes that originate in mitochondrial dysfunction, often as a consequence of variants in the mitochondrial DNA (mDNA) or nuclear DNA (nDNA). Although many variants and related phenotypes are rare, as a group they form one of the more common genetic disorders with a minimum prevalence rate of 1:5000 [13]. Any organ or tissue throughout the human body can be affected, although the organs that are most dependent on the production of energy by the mitochondria (e.g. brain, skeletal muscles) are most frequently and most severely affected [8, 33]. As a result, mitochondrial defects are associated with a broad spectrum of clinical manifestations [2].

The heterogeneous and often severe nature of MD has been shown to impact patient quality of life (QoL) [15, 37]. Furthermore cognitive impairments [7, 20, 26], mental health problems [9, 17, 20, 23, 37], and fatigue [12, 30, 37] have been demonstrated to be common in patients with MD. Despite the evident impairments that are associated with MD, studies on relationships between biological and physiological aspects of MD, patient reported symptoms, societal participation and QoL are scarce. More information on these relationships could shed light on factors contributing to patient reported symptoms, societal participation and QoL next to specific disease related factors. QoL is only partly reflected by clinical parameters and symptom status, as shown by the study of Verhaak et al. [37]. This is in line with other studies showing several factors contributing to QoL in chronic diseases, amongst which for example depressive symptoms, cognitive impairment, and health risk behaviors [24].

Although information on the subject is still limited, QoL is important as an outcome measure in chronic disease research, and information from patient reported outcomes regarding QoL is essential to provide adequate support and tailored care for patients. Adequately investigating the causal factors and mediators in QoL requires proper conceptualization of disease aspects and the use of models rather than only descriptive methods. The Wilson and Cleary model [40] is the most widely cited conceptual framework to assess QoL in chronic diseases [3]. This model describes a causal pathway of several determinants, in terms of different levels of health concepts, in QoL. These include: (1) The biological and physiological factors: amongst which cell functioning and organs; (2) Symptom status: the patient’s own perception of their cognitive, physical or emotional functioning; (3) Functional health: the physical, psychological, social and role functioning; (4) General health perceptions: subjective rating of an integrated concept of all health aspects; (5) Overall quality of life: subjective estimate of the patients overall well-being. Information from this model could give directions for interventions in patients with MD [19, 29]. The relationships between the various levels of health concepts that contribute to QoL in patients with MD remain complicated and should be further investigated. Based on Wilson and Cleary’s model, we adapted these health concepts for MD including: (1) Biological and physiological level: genotype and phenotype, heteroplasmy, levels of the mDNA variants, and disease manifestation; (2) Symptom status: cognitive functioning, fatigue and depressive symptoms; (3) Functional health: societal participation; (4) Patient reported health perceptions: QoL related physical functioning, mental health, and social functioning; (5) Overall QoL.

The aim of this study was to investigate the interrelationships between biological and physiological functioning, cognitive functioning, mental health, fatigue, societal participation and health perceptions, and QoL. The Wilson and Cleary model was adapted to MD and used as a concept to provide information on factors contributing to QoL. Studying the relationships between these various determinants is essential in providing adequate support and directions for patient care. Furthermore, it could provide a framework for the development of interventions, treatments and clinical trials in the future.

## Methods

### Sample and procedure

Eligible patients were age 18 or older, diagnosed with MD as caused by variants in mDNA or nDNA and followed-up by the Radboud Center for Mitochondrial Medicine (RCMM).

The RCMM is an internationally recognized center of expertise, focused on diagnosis, clinical care and research for patients with MD. ‘The Mitolane’ is a multidisciplinary admission that is run within the RCMM, in which various organ systems and functioning of patients with MD are structurally evaluated and monitored. Patients filled out various questionnaires before admission. Data derived from these questionnaires were used in this study. This study was approved by the local ethical committee of the Radboud University Medical Centre, Nijmegen, The Netherlands (2017-3687). All patients gave informed consent.

### Outcome measures

**Fig. 1:**
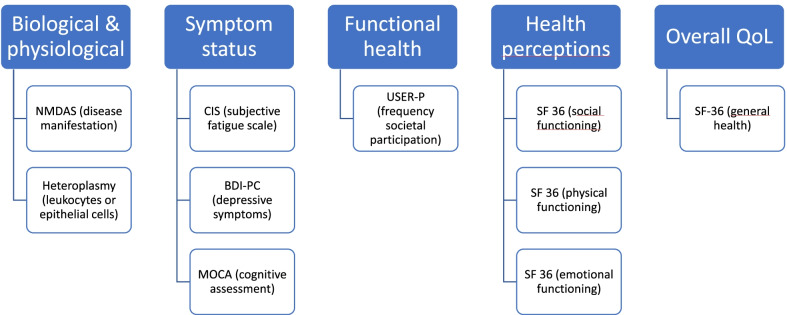
Schematic overview of the Wilson and Cleary model adapted to MD

Assessments were based on application of The Wilson and Cleary model [40], adapted to MD and discussed by experts from the RCMM until consensus was reached (see Fig. 1).

#### Biological and physiological variables

*Genotype* of mitochondrial DNA was assessed in terms of *heteroplasmy* levels in leukocytes or urine epithelial cells [22]. Genotype of nuclear DNA was determined by a polymerase chain reaction-test (PCR) and sequence analysis of the gene or by whole exome sequencing.

Every patient was categorized into one of the following clinical phenotype groups: (1) MELAS syndrome, if stroke-like episodes (SLE) or signs or significant encephalopathy were present. (2) MIDD syndrome, if the patient had glucose intolerance and hearing loss, but no SLE or significant encephalopathy. (3) MERFF syndrome MERRF (Myoclonic Epilepsy with Ragged-Red Fibers. (4) The remaining symptomatic patients were classified as ‘other’. This group consisted of patients with isolated, sometimes severe, cardiomyopathy and patients with nephropathy leading to end stage renal failure requiring transplantation. In addition, this group contained patients with only hearing loss or diabetes, who do not or not yet qualify for the MIDD diagnosis, and patients with isolated myopathy and/or significant fatigue. *Disease manifestation* was assessed by the Newcastle Mitochondrial Disease Adult Scale (NMDAS). The NMDAS is a validated method for both measurement and monitoring of disease manifestation and clinical features in patients with MD [32] and consists of three sections: section I: current functioning, section II: system specific involvement and section III: current clinical assessment. All questions can be scored from 0 (no involvement) to 5 (severe involvement). Higher scores on the NMDAS and its subscales are associated with higher symptom severity and clinical manifestation of the disease. Patients were divided into three disease manifestation subgroups based on the total NMDAS score: mild (0–10), moderate (11–20) and severe (> 20).

#### Symptom status

*Cognitive assessment* was performed using the Montreal Cognitive Assessment (MoCA) [28]. The MoCA is a brief tool that is used for the screening of mild cognitive impairment (MCI), by assessing various cognitive abilities. This includes visuospatial/executive functioning, naming, episodic memory, attention, language, abstraction, and orientation. It has been found to be valid among a wide range of disorders that present with cognitive impairment [18]. The MoCA is scored out of 30 points, with a lower score being associated with more cognitive impairment. A score of 26 or above is indicative of normal cognitive functioning, whereas scores of 25 or lower are labelled as ‘suspected of MCI’.

*Depressive symptoms* were assessed using the Beck Depression Inventory (BDI) [5]. BDI is a screening device for major depressive disorder in psychiatric and non-psychiatric subjects. The BDI score is reliant on physical symptoms. Scores may therefore be inflated in patients with symptoms such as fatigue. For this reason, the Beck Depression Inventory for Primary Care (BDI-PC) was used in this study. The BDI-PC is a short screening method that does not focus on physical symptoms and is therefore more reliable in patients with MD. Each of the 7 questions on the BDI-PC yields a score from 0–3, resulting in a total score between 0 and 21. A cut-off score of 4 was used to differentiate between not depressed (0–3) and depressed (≥ 4) [4].

*Fatigue severity* was assessed using the Checklist Individual Strength (CIS) [36]. The ‘subjective fatigue’ subscale was used for this study since it measures fatigue as perceived by the patient. This scale contains 8 items (scores between 8 and 56). The CIS can be scored on a seven-point Likert scale, ranging from ‘yes, that is correct’ to ‘no, that is incorrect’, a higher score on the checklist is associated with worse fatigue. Scores of > 35 on the subjective fatigue subscale were rated as severe fatigue [36, 41].

#### Functional health

*Societal participation* was assessed using the Utrecht Scale for Evaluation of Rehabilitation-Participation (USER-P) [31]. The USER-P was designed to measure both objective and subjective participation to provide an accurate measurement of societal participation and has been studied on terms of validity, reproducibility and effectivity, as compared to other measures of participation [31, 42, 43]. It is comprised of 31 items, divided over three scales. The Frequency scale (scored from 0 to 5) measures the time per week spend on paid work, unpaid work, volunteer work, housekeeping, leisure and social activities. The Restrictions scale (scored from 0 to 3) measures participation restrictions in vocational, leisure and social domains. The Satisfaction scale (scored from 0 to 4) evaluates personal satisfaction with vocational, leisure and social domains. All three scales individually generate a sum score, which are transformed into a 0–100 scale for analyses. A higher score indicates better societal participation.

#### Patient reported health perceptions

*Quality of life* was assessed by the SF-36 health survey [39, 38]. SF-36 is a 36-item questionnaire regarding patient reported health and quality of life. The SF-36 describes eight health concepts: physical functioning, bodily pain, role limitations due to physical health problems, role limitations due to personal or emotional problems, mental health, social functioning, vitality, and general health perceptions. All items were individually scored on a 0–100 scale and averaged together to create the subscale scores that were used in analysis. A higher score on the SF-36 indicates a more favourable health outcome in that domain. The subscales physical functioning, mental health, and social functioning were used for the patient reported health perceptions. For the assessment of the overall QoL the subscale general health perceptions of the SF-36 was used.

### Data analysis

Data analysis was performed using SPSS version 26.0. Logarithmic transformations were used to transform variables with a skewness larger than 1 or smaller than -1. One sample t-tests and Wilcoxon signed rank tests were performed to compare results to data of healthy norms and other patient groups.

Scores of patients with MD were compared to normative data and/or other patient groups, based on reference groups available for different instruments. The mean BDI-PC total score was compared to a group of medical inpatients referred to psychiatric consultation [4], as well as to a group of medical outpatients who were seen by a medical specialist for one or multiple medical disorders like cardiovascular or respiratory disease [34]. Mean scores of the CIS were compared to normative data of healthy controls, patients with multiple sclerosis (MS) and patients with chronic fatigue syndrome (CFS) [36]. Scores of the USER-P scales were compared to a combined cohort of patients with musculoskeletal disease, brain injuries, neurological diseases, heart conditions and chronic pain [43] and a group of stroke patients [44]. Results of the SF-36 questionnaire were compared to a Dutch norm group [35].

Pearson correlation analyses were used to, univariately, investigate the correlations between the included variables. Correlations between 0.70 and 0.90 were considered strong, between 0.50 and 0.70 moderate, 0.30 and 0.50 low, and below 0.30 were interpreted as little if any correlation [16].

A hierarchical regression analysis was performed to explore the predictive value of the different steps contributing to the QoL according to the adapted Wilson and Cleary’s conceptual disease model. Prediction of the overall QoL was multivariate investigated by the previous levels of health concepts according to the model were entered as separated steps. The first step in the model was NMDAS, next symptom status (MOCA, CIS and BDI-PC) was entered. Next functional health (USER-P frequency), and finally the health perceptions (SF-36 physical, emotional and mental health subscales) were entered in the regression analysis. Since the level of heteroplasmy in leucocytes and/ or urine epithelial cells was only available for a small number of patients, we did not include this variable in the hierarchical regression analyses. Demographic variables including age, education and sex, were not entered in the regression analyses based on an absence of a correlation with the included variables in the model as assessed in this study and due to limited power.

## Results

### Response

Of all eligible patients in the Mitolane, 95 filled in all relevant questionnaires upon admission into the RCMM. Patients that were not present for every test or that failed to completely fill out each questionnaire were excluded only for the specific test or questionnaire in which response was missing, leading to different response rates for NMDAS (97.9%), MoCA (86.3%), BDI (96.8%), CIS (98.9%), SF-36 (98.9%), USER-P (95.8%).

### Demographics, biological and physiological variables

See Table 1 for patient characteristics. Most patients carried the mDNA 3243A > G variant (60%). The other 40% consisted of multiple small groups with different variants. All included variants are presented in Additional file 1: Table S1. Patients were divided into three disease manifestation categories, based on the NMDAS total score: mild (16.1%), moderate (41.4%) and severe symptoms (42.3%).

**Table 1:** General patient characteristics (N = 95), phenotype, disease manifestation (NMDAS)

	% (N/total)	Mean	SD	Median	Range
Age (years)		43	12.3	41	19–70
Gender male	33.6 (32/95)				
Heigth (cm)		1.70	0.1	1.68	1.45–1.89
Phenotype					
MELAS	11.6 (11/95)				
MIDD	43.2 (41/95)				
MERRF	10.5 (10/95)				
Others	34.7 (33/95)				
NMDAS					
I current functioning		10	7	7.5	0–33
II system specific involvement		7	4	7	0–26
III current clinical assessment		5	4	4	0–16
Total score		21.66	12.1	19	3–69

### Symptom status

#### Cognitive functioning

The mean of the MoCA total score was 26 (SD 3), with scores ranging from 17 to 30. One-third of the patients (35.4%) scored below the cut-off score of 26, indicative for suspicion of mild cognitive impairment (MCI).

#### Depressive symptoms

A total of 26 patients (27.4%) scored above the cut-off score for depression. The patients scored significantly lower compared to medical inpatients (t = − 10.243; *p* < 0.001) [4], yet comparable to the outpatient group (t = 1.809,* p* = 0.074) (mean total score patients 2.7 (SD 2.8)) [34].

#### Fatigue

Based on the subjective fatigue subscale, 79.8% of the patients experienced severe fatigue. Patients experienced significantly more fatigue compared to healthy norms (t = 25,684; *p* < 0.001) and MS patients (t = 2.245; *p* = 0.027), yet scored significantly lower compared to CFS patients (t = − 9.526; *p* < 0.001).

### Functional health

Mean scores on the USER-P Frequency, Restrictions and Satisfaction subscales were 31.8, 72.4 and 59.6 respectively. Frequency and Restrictions scores were not significantly different in patients with MD compared to the patients with musculoskeletal disease, brain injuries, neurological diseases, heart conditions and chronic pain. However, patients with MD scored significantly lower on the Satisfaction subscale (t = − 2.127; *p* = 0.036). Furthermore, patients with MD scored significantly higher on the Frequency scale compared to stroke patients (t = 5.328; *p* < 0.001), yet lower on the Restrictions scale (t = − 3.612; *p* < 0.001) and Satisfaction scale (t = 6.411; *p* < 0.001).

### Patient reported health perceptions and overall quality of life

The results of the *SF-36 questionnaire* showed that patients with MD scored significantly lower compared to the norm group in all SF-36 subscales (Table 2).

**Table 2:** Quality of life subscale scores (SF-36) of patients and healthy norms

SF-36 subscales	Mean	SD	Norm	SD	t-value	*p* value
Physical functioning	56.8	26.0	81.9	23.2	− 9.362	< 0.001
Role limitations due to physical health problems	30.9	36.6	79.4	35.5	− 12.876	< 0.001
Role limitations due to emotional problems	70.2	38.9	84.1	32.3	− 3.457	0.001
Mental health	69.0	18.1	76.8	18.4	− 4.190	< 0.001
Vitality	43.0	17.5	67.4	19.9	− 13.517	< 0.001
Social functioning	60.9	23.1	86.9	20.5	− 10.908	< 0.001
Pain	61.5	21.4	79.5	25.6	− 8.137	< 0.001
General health	31.7	17.0	72.7	22.7	− 23.481	< 0.001

A higher score indicates a better QoL

*p < 0.05; **p < 0.001

### Correlation analyses

The demographic variables age and gender, only showed little if any correlations with some of the biological and physiological variables. There was no significant correlation between level of heteroplasmy as measured in urine and leucocytes and the NMDAS. The NMDAS was low to moderately correlated with cognitive functioning, societal participation and physical functioning, and a low correlation with overall QoL. Almost all the PRO’s between the remaining steps (symptom status, functional health, health perceptions, and overall QoL) showed a low to moderate, but significant correlation (Table 3).

**Table 3:** Pearson correlations between the 5 levels of health concepts

	(1)	(2)	(3)	(4)	(5)	(6)	(7)	(8)	(9)	(10)	(11)	(12)	(13)	(14)
*Demographics*														
(1) Age	1.00													
(2) Gender	− 0.281**	1.00												
(3) Education	0.324**	− 0.199	1.00											
*Biological and physiological*														
(4) Heteroplasmy urine	− 0.203	− 0.298*	0.005	1.00										
(5) Heteroplasmy leucocytes	− 0.278*	0.273*	− 0.062	0.387**	1.00									
(6) Disease manifestation (NMDAS)	0.021	− 0.253*	0.027	0.132	0.052	1.00								
*Symptom status*														
(7) Cognitive functioning (MOCA)	− 0.138	0.161	0.039	0.011	− 0.006	− 0.519**	1.00							
(8) Fatigue (CIS F)	− 0.006	− 0.063	0.153	− 0.129	0.054	0.053	0.045	1.00						
(9) Depression (BDI-PC)	− 0.132	− 0.053	− 0.122	− 0.015	0.014	0.123	− 0.074	0.342**	1.00					
*Functional health*														
(10) Societal participation (USERP F)	− 0.025	0.173	0.037	0.079	0.029	− 0.395**	0.593**	− 0.322**	− 0.304**	1.00				
*Health perceptions*														
(11) QoL physical functioning (SF36 PF)	− 0.184	0.146	− 0.163	0.316*	0.027	− 0.545**	0.313**	− 0.418**	− 0.141	0.411**	1.00			
(12) QoL mental health (SF36 MH)	0.068	0.009	0.003	0.038	− 0.020	0.058	0.084	− 0.371**	− 0.813**	0.315**	0.039	1.00		
(13) QoL social functioning (SF36 SF)	0.005	0.026	− 0.010	0.238	− 0.065	− 0.161	0.223*	− 0.453**	− 0.611**	0.437**	0.338**	0.723**	1.00	
*Overall QoL*														
(14) QoL general health (SF36 GH)	0.051	0.084	− 0.082	0.182	− 0.074	− 0.299**	0.089	− 0.578**	− 0.376**	0.386**	0.461**	0.403**	0.488**	1.00

Bold values indicate significance results

*Correlations are significant with *p* < 0.05

**Correlations are significant with *p* < 0.01

*p < 0.05; **p < 0.01

### Prediction of the overall QoL

See for a detailed overview of the specific hierarchical regression analysis Table 4.

**Table 4:** Hierarchical regression model overall QoL

	Outcome measure	Bivariate regression coefficient	Multivariate regression coefficient	
		Correlation with QoL general health (SF 36 GH)	Regression coefficient (B (95% CI))	Standardized coefficient (Beta)
	Constant		55.32 (13.07 to 97.56)	
Biological and physiological	Disease manifestation (NMDAS)	− 0.34	− 0.03 (− 0.69 to 0.09)	− 0.19
Symptom status	Depression (BDI-PC)	− 0.34	0.38 (− 1.46 to 2.22)	0.06
	Fatigue (CIS F)	− 0.59	− **0.64 (**− **1.02 to** − 0.27)**	− 0.36
	Cognitive functioning (MOCA)	0.09	− 0.73 (− 2.08–0.63)	− 0.13
Functional health	Societal participation (USERP F)	0.36	0.05 (− 0.33–0.42)	0.03
Health perceptions	QoL physical functioning (SF36 PF)	0.48	**0.17 (0.01**–0.33)*	0.08
	QoL mental health (SF36 MH)	0.34	0.20 (− 0.14–0.54)	0.20
	QoL social functioning (SF36 SF)	0.56	0.06 (− 0.14–0.26)	0.26

Bold values indicate significance results

*p < 0.05; **p < 0.01

Results of the hierarchical regression analysis showed that the *overall quality of life* was significantly predicted by fatigue and physical functioning. Disease manifestation, functional status and health perceptions did not add significant explained variance. The total regression model explained together 71.1% of the variance.

## Discussion

The aim of this study was to investigate the status of and interrelationships between health concepts: biological and physiological functioning, symptom status, functional health, health perceptions and QoL by using the Wilson and Cleary conceptual model adapted to MD. Results underlined severe disease manifestation, cognitive impairments, severe fatigue, depressive symptoms, impairments in societal participation and significant QoL impairments in patients with MD. In univariate analyses, disease manifestation was only significantly correlated with cognitive functioning, participation and physical functioning, while symptom status variables (in terms of cognitive functioning, fatigue, and depression) were correlated with almost all outcome measures regarding functional health, health perceptions and QoL. Multivariate assessments showed that the overall QoL was significantly predicted by fatigue and physical functioning, and remarkably, disease manifestation did not explain significant variance in overall QoL. Results will be discussed following the levels of health concepts according to the adapted Wilson and Cleary model.

Regarding the *biological and physiological level*, results showed that most patients experienced moderate (41%) to severe (42%) clinical symptoms as measured by the NMDAS. Contrary as expected based on previous studies [22], this study did not find a significant correlation between disease manifestation and levels of heteroplasmy. Although heteroplasmy as measured in blood is highly correlated with disease burden as measured in patients with the mDNA 3242A > G variant [14], we did not find a relation with heteroplasmy as measured in urine nor in leucocytes. A possible explanation might be our study contained a heterogeneous sample with 60% of the patients carrying the mDNA 3243A > G variant, and the remaining 40% consisted of 19 different genetic variants. In every variant, symptoms arise at different threshold levels. This might explain the absence of a correlation when investigating the different genetic variants as one group. This in contrast to the study of De Laat et al. [22], in which all patients carried the mDNA 3243A > G variant and also carriers were included. Furthermore, the disease manifestation in this study was slightly higher compared to other studies (median overall NMDAS [26, 27] score of 19 compared to 16 in Verhaak et al. [37], and 15 in the study of De laat et al. [22], most likely explained by differences in included MD. It is known that the disease course, the expression of the disease, and its manifestation, are heterogenous and can vary between but also within different geno- and phenotypes [6, 11].

The relationship between disease manifestation and cognitive impairments was the only significant correlation between the *biological and physiological level* and *symptom status*. No relationship between disease manifestation and fatigue or disease manifestation and depressive symptoms was found. Regarding cognitive functioning, impairments are common in MD as several review studies showed [20, 26]. In this study, 35.4% of the patients showed cognitive impairments indicative of MCI. In line with previous review studies [21, 26], more cognitive impairments were related to a higher disease manifestation. Results underline the importance of screening for cognitive impairments by an increasing disease manifestation. Regarding fatigue, results of this study showed that almost 80% of the patients experienced severe fatigue. This is in line with previous studies in MD, reporting fatigue in 60–100% of the patients [12, 30, 37]. The absence of a relation between disease manifestation and fatigue is contrary as expected based on other studies, however those studies solely investigated patients with the mDNA 3243 A > G variant [30, 37]. In line with this study, the study of [12, 13] investigated a heterogeneous group of MD, and reported a significant correlation between fatigue and NMDAS on some domains, e.g. activities of daily living, but not on the domains of for example muscle weakness or severity of encephalopathy. They did not report a relation between genotype, eliminating a possible explanation for differences in results between studies. Based on this, it is suggested that fatigue is a common and severe complaint in MD, however its relation with disease manifestation is not straightforward. Although fatigue is one of the core symptoms of MD, and there is a direct link with mitochondrial functioning, research on other chronic diseases show that generic factors also can influence fatigue, like cognitions and behaviors [1, 20, 25]. Whether cognitive behavioral therapies are effective in MD has yet to be investigated. At this moment, our research group is performing a single case experiment, a cognitive behavioral therapy targeting fatigue, in children and adolescents with MD [20]. Regarding depressive symptoms, more than a quarter (27.4%) of the patients were positively screened for depressive symptoms. Although this was lower compared to psychiatric patients, it was comparable to patients with various other diseases, who had three medical diagnoses in almost half of the cases, indicating a severe medical condition. Another study on MD reported an estimated lifetime prevalence of 54% for major depressive disorder [10]. The percentage in the current study was considerably lower, however depression was only screened for presence of depressive symptoms at the time of assessment and not as a lifetime prevalence. The absence of a relation between disease manifestation and depression, is in line with results of Parikh et al. [30]. As expected results showed a small but significant correlation between fatigue and depressive symptoms, in line with results of Parikh et al. [30] and Verhaak et al. [37] although the latter included a combination of anxiety and depressive symptoms. It is still unclear if increased fatigue is a cause of increased depressive symptoms, or the other way around. However, the high incidence of fatigue and depressive symptoms, their correlation, and the absence of a link with disease manifestation, making routine assessments and appropriate therapy for these symptoms in patients with MD necessary.

Regarding *functional health status*, the frequency and restrictions of societal participation were comparable to other chronic diseases [43], however patients reported to be less satisfied with their daily activities. These results possibly indicate that although the amount of activities and experienced restrictions are comparable to other chronic diseases, patients with MD want to do more than they are capable of. Unfortunately, there was no comparison data available for specific chronic diseases. This study did not include any measure of disease acceptation or coping with the disease, which can be of interest in light of these results. There was a significant negative correlation between the frequency of societal participation and disease manifestation, indicating that patients with a higher disease manifestation, participated less in society. Furthermore, there was a significant correlation with each of the symptom status variables; higher levels of fatigue, more symptoms of depression, and more cognitive impairments were related to less frequent societal participation. These results give directions for interventions targeting fatigue, cognitive functioning or coping skills and could influence participation in society.

Regarding *health perceptions* and *overall QoL,* in line with previous studies [37], results showed a worse QoL on all domains compared to normative data. Regarding the health perceptions, a higher disease manifestation was only related with a worse physical functioning, but not related with mental health or social functioning. Higher levels of fatigue and less participation in society were related to less favorable health perceptions on all domains. Furthermore, depressive symptoms were correlated with mental health and social functioning, while cognitive impairments were correlated with both physical and social functioning. These results indicate that all symptom status variables are of influence on the health perceptions, though the individual effects are reflected on different domains.

Investigating the factors contributing to QoL, this study showed that the overall quality of life was only predicted by fatigue and physical QoL. Despite significant correlations with all of the health concepts according to the Wilson and Cleary model, after controlling for the different health concepts, these did not contribute to the overall QoL. These results indicate that in order to optimize QoL, fatigue and physical QoL are important factors to be aware of. As mentioned before, these results give directions for screening and interventions, and furthermore highlight the need to look beyond medical aspects to optimize patient care.

As hypothesized in the original Wilson and Cleary model [40] there are causal links between the different levels of health concepts: biological and physiological level, symptom status, functional status, general health perceptions and QoL. This model has been proven useful in identifying relationships among these health levels, and predictors of QoL in different disease populations [29]. Results from Ojelabi et al. [29] show that symptoms are a major determinant of QoL in patients with chronic disease, and suggest that reducing symptoms, especially depression, may improve QoL. Results from this study underline the importance of symptoms in relation to health perceptions and overall QoL. Based on this study, the Wilson and Cleary model seems useful in MD.

A limitation of this study is its cross-sectional design. In order to analyse causes and consequences, a longitudinal design is preferred. However, by using an adapted version of the Wilson and Cleary model, the different steps in the model were based on theory, making predictions possible. For future research, longitudinal studies investigating patients from start of diagnosis on a regular basis is recommended to investigate the model in relation to the disease course. This study included a heterogeneous sample of MD, instead of one genotype. A limitation of this is the generalizability to specific genotypes, however by including all types of MD, more generic disease factors can become visible. Since most patient care standards do not (yet) differentiate between different types of MD, it is important to investigate all types of MD and not eliminate rare subtypes. Furthermore, by obtaining a broad view, directions for future research can be made.

In conclusion, based on an adapted version of the Wilson and Cleary conceptual model, results of this study showed that symptom status (in terms of cognitive functioning, fatigue, and depression) is related to the functional health, health perceptions and QoL. Moreover, in the total model fatigue and physical functioning are important contributors to the overall QoL of MD patients. Remarkably, disease manifestation did not explain significant variance in overall QoL. In order to provide adequate patient care it is therefore important to have a broad view on the patients functioning, not only by providing a proper clinical assessment, but also by screening for patients symptoms regarding fatigue, cognitive functioning and depression. As highlighted in this study, screening for these symptoms can give therapeutic directions to optimize patient care.

## Supplementary Information

**Additional file 1. Table S1.** Included mutations.

## Data Availability

The datasets analyzed during the current study are available from the corresponding author on reasonable request.

## Abbreviations

MD: Mitochondrial disease
RCMM: Radboud Center for Mitochondrial Medicine
QoL: Quality of life
NMDAS: Newcastle Mitochondrial Disease Adult Scale
MoCA: Montreal Cognitive Assessment
BDI: Beck Depression Inventory
USER-P: Utrecht Scale for Evaluation of Rehabilitation-Participation
CIS: Checklist Individual Strength
MCI: Mild cognitive impairment
SD: Standard deviation
