# Supplementary material for: A conceptual disease model for quality of life in mitochondrial disease
Source: Orphanet J Rare Dis. 2022 Jul 15;17:263. doi: 10.1186/s13023-022-02411-9 (PMC9287990; doi:10.1186/s13023-022-02411-9)
Supplement: Supplementary file 1 — Additional file 1. Table S1. Included mutations. [file 13023_2022_2411_MOESM1_ESM.docx]

**Supplementay table 1:** included mutations

| Mutation | N | % |
| --- | --- | --- |
| mDNA3243 A>G | 57 | 60 |
| mDNA8363 A>G | 6 | 6.3 |
| m.8344 A>G | 5 | 5.3 |
| mDNA deletion | 4 | 4.2 |
| mDNA13513 G>A | 3 | 3.2 |
| OPA1 | 3 | 3.2 |
| mDNA14484 T>C | 2 | 2.1 |
| mDNA10191 T>C | 2 | 2.1 |
| mDNA7471 ins. C | 2 | 2.1 |
| c.1120 C>T (Twinkle gene) | 1 | 1.0 |
| GTPB3 | 1 | 1.0 |
| c.1594 C>T (ACAD9) | 1 | 1.0 |
| mDNA3173 G>A | 1 | 1.0 |
| mDNA7495 A>G | 1 | 1.0 |
| mDNA9155 A>G | 1 | 1.0 |
| mDNA3271 T>C | 1 | 1.0 |
| mDNA3460 G>A | 1 | 1.0 |
| mDNA8993 T>G | 1 | 1.0 |
| mDNA9176 T>C | 1 | 1.0 |
| MTO1 | 1 | 1.0 |
